# TGFβ in malignant canine mammary tumors: relation with angiogenesis, immunologic markers and prognostic role

**DOI:** 10.1080/01652176.2024.2390941

**Published:** 2024-08-20

**Authors:** Maria Isabel Carvalho, Ricardo Silva-Carvalho, Justina Prada, Carla Pinto, Hugo Gregório, Luis Lobo, Isabel Pires, Felisbina L. Queiroga

**Affiliations:** a MVET Research in Veterinary Medicine. Faculty of Veterinary Medicine, Lusófona University – Lisbon Centre, Lisboa, Portugal; b CEB – Centre of Biological Engineering, University of Minho, Braga, Portugal; c LABBELS – Associate Laboratory, Braga, Guimarães, Portugal; d Veterinary and Animal Research Center (CECAV), University of Trás-os-Montes and Alto Douro, Vila Real, Portugal; e Department of Veterinary Sciences, University of Trás-os-Montes and Alto Douro, Vila Real, Portugal; f Anicura Centro Hospitalar Veterinário, Porto, Portugal; g Onevet Hospital Veterinário do Porto, Porto, Portugal; h Center for the Study of Animal Sciences, CECA-ICETA, University of Porto, Portugal

**Keywords:** Angiogenesis, canine mammary tumors, CD31, FoxP3, prognosis, TGFβ, Treg cells, VEGF

## Abstract

Transforming growth factor-β (TGFβ) and FoxP3 regulatory T cells (Treg) are involved in human breast carcinogenesis. This topic is not well documented in canine mammary tumors (CMT). In this work, the tumoral TGFβ expression was assessed by immunohistochemistry in 67 malignant CMT and its correlation to previously determined FoxP3, VEGF, and CD31 markers and other clinicopathologic parameters was evaluated. The high levels of TGFβ were statistically significantly associated with skin ulceration, tumor necrosis, high histological grade of malignancy (HGM), presence of neoplastic intravascular emboli and presence of lymph node metastases. The observed levels of TGFβ were positively correlated with intratumoral FoxP3 (strong correlation), VEGF (weak correlation) and CD31 (moderate correlation). Tumors that presented a concurrent high expression of TGFβ/FoxP3, TGFβ/VEGF, and TGFβ/CD31 markers were statistically significantly associated with parameters of tumor malignancy (high HGM, presence of vascular emboli and nodal metastasis). Additionally, shorter overall survival (OS) time was statistically significantly associated with tumors with an abundant TGFβ expression and with concurrent high expression of TGFβ/FoxP3, TGFβ/VEGF, and TGFβ/CD31. The presence of lymph node metastasis increased 11 times the risk of disease-related death, arising as an independent predictor of poor prognosis in the multivariable analysis. In conclusion, TGFβ and Treg cells seem involved in tumor progression emerging as potential therapeutic targets for future immunotherapy studies.

## 1. Introduction

Transforming growth factor-β (TGFβ), a multitasking cytokine expressed in a variety of tissues, exert its activities through 2 serine-threonine kinases receptors: TGFβRI and TGFβRII (Derynck et al. 2001; Sigal 2012; Principe et al. 2014; Hu et al. 2018). Once the ligand is activated, TGFβ signaling is mediated through SMAD and non-SMAD pathways. The SMAD signaling pathway requires the phosphorylation and subsequent translocation of SMAD complexes to the nucleus where it interacts with transcriptional co-regulators and other factors to mediate target gene expression or repression (Shi and Massagué 2003; Hata and Davis 2009; Hu et al. 2018; Tzavlaki and Moustakas 2020). Although less frequent, the non-SMAD pathways contribute to cell proliferation, motility, and survival through p38 MAPK, p42/p44 MAPK, Rho GTPase, PI3K/Akt signaling activation (Hong et al. 2011; Mu et al. 2012).

TGFβ actively participate in key biological functions related to homeostatic cellular pathways (including apoptosis, proliferation and immunity) (Flanders et al. 2016), and is critically important for mammary morphogenesis and secretory function through specific regulation of epithelial proliferation, apoptosis, and extracellular matrix (Moses and Barcellos-Hoff 2011). Nevertheless, increasing evidence suggests that TGFβ signaling plays also an important role in malignant transformation in breast cancer, participating in cancer cell migration, survival and angiogenesis (Gupta et al. 2007; Moses and Barcellos-Hoff 2011; Chen et al. 2016; Ding et al. 2016; Zhao et al. 2018).

TGFβ demonstrates a paradoxical role in malignant mammary tumor process. In early stages of carcinogenesis, this cytokine seems to restrain growth and serves as a tumor suppressor. However, with the development of malignancy, TGFβ becomes a promoter of tumor cell invasion and metastasis (Dumont and Arteaga 2000; Bierie and Moses 2009; Principe et al. 2014; Colak and Ten Dijke 2017).

For instance, the dysregulation of TGFβ pathways in breast cancer have been correlated with disease progression, allowing cancer cells to warrant their own survival (Dumont and Arteaga 2000; Chen et al. 2016; Juang et al. 2016; Xie et al. 2018). Furthermore, TGFβ seems to shape the tumor microenvironment and, when produced in excess by tumor cells, act in a paracrine manner on the peritumoral stroma, tumor neovessels and immune system resulting in increased cell–matrix interaction and angiogenic activity and suppressed immune surveillance which fosters tumor development (Gorsch et al. 1992; Bao et al. 2009; Lang et al. 2014; Ding et al. 2016; MaruYama et al. 2022).

By avoiding the tumor-suppressive roles of TGFβ, mammary cancer cells can take advantage of its potent immunosuppressive functions. For instance, TGFβ signaling in T cells represses both their inflammatory and cytotoxic differentiation programs (Dumont and Arteaga 2000; Padua and Massagué 2009; Liu et al. 2020; van den Bulk et al. 2021; MaruYama et al. 2022). In addition to impairing T cells effector functions, TGFβ plays a pivotal role in the generation of regulatory T cells (Tregs) from a population of peripheral CD4^+^CD25^-^T cells through the induction of the key transcription factor FoxP3 (Fantini et al. 2004; Chen and Konkel 2010). In human breast cancer, TGFβ and FoxP3 share signaling pathways with a crucial impact in several tumor hallmark steps, including angiogenesis, facilitating nutrient exchange and metastasis (Gupta et al. 2007; Padua and Massagué 2009; Chen and Konkel 2010; Wang et al. 2013; Lainé et al. 2021). Both TGFβ and FoxP3 are reported to be sufficient to upregulate the expression of vascular endothelial growth factor (VEGF), one of the most selective and potent angiogenic factors known, attracting adjacent endothelial cells and promoting the formation of tumor neovascularization (Donovan et al. 1997; Gupta et al. 2007; Kajal et al. 2021).

In human breast cancer, the role of TGFβ among the different tumor sub-types has been a subject of interest. TGFβ seems to have a tumor suppressor effect mainly in luminal breast cancer and initial stages of tumors. On the other hand, in HER2^+^ and triple negative sub-types seems to have a pro-tumorigenic effect (Tang et al. 2003; Wilson et al. 2005; Parvani et al. 2011). In a recent study, Vitiello et al. (Vitiello et al. 2021) suggested that TGFβ signaling exert tumor-suppressive effects in luminal-B-HER2^+^ and p53-negative in breast cancers. Additionally, in humans TGFβ and FoxP3 have an active role in the VEGF signaling and in tumor angiogenic switch by promoting an increased intratumoral microvessel density, which contributes to mammary carcinogenesis and poor prognosis (Gupta et al. 2007; Kajal et al. 2021).

Regarding canine mammary tumors (CMT), some contradictory studies were published (Klopfleisch et al. 2010; Yoshida et al. 2013). An *in vitro* study using a mammary gland tumor cell line (CHMp13a) suggested that TGFβ induces invasiveness capacity of the cells (Yoshida et al. 2013). These findings do not support those of Klopfleisch et al. (2010), who reported that increased tumoral proliferative activity was related to a loss of TGFβ-3 and LTBP-4 coupled with reduced TGFβR-3 expression. Furthermore, Treg cells seems to play a role in CMT development and aggressiveness and may contribute to increased angiogenesis (Carvalho et al. 2016). Another *in vitro* study showed an increase in FoxP3 mRNA and protein expression in activated dog lymphocytes stimulated with TGFβ and IL-2. Despite less prominent, tumor cell receptor activation alone induced small increases in FoxP3 expression. All of these results suggest that the regulation of FoxP3 expression in dog and human Tregs is similar (Biller et al. 2007). However, to the best of our knowledge the prognostic value and the correlation between TGFβ and FoxP3 Treg cells expression in dog mammary tumors has not been investigated yet.

To elucidate the potential association of TGFβ and FoxP3 with angiogenesis and clinical outcome in malignant CMT, immunohistochemistry was performed to detect the expression of TGFβ in a series of malignant CMT. We also aimed to assess the correlation between the expression of TGFβ with intratumoral FoxP3 Treg cells, and angiogenesis markers [VEGF expression, microvessel density (MVD)] previously determined in the same tumors and published (Carvalho et al. 2016). Furthermore, 2 years follow up of the dogs enrolled in this study was performed to determine the overall survival rate.

## 2. Materials and methods

### 2.1. Sample selection and clinicopathological analysis

A total of 67 female dogs of different breeds, with malignant mammary tumors received for diagnosis and treatment, were included in this study. As reported in our previous study (Carvalho et al. 2016), all animals (mean age of ∼10 years) were free from distant metastasis at the time of diagnosis (confirmed throughout thorax X-ray and abdominal ultrasound) and were only submitted to surgery (regional or complete mastectomy) as treatment (chemotherapy and/or radiation therapy was not performed). For the analysis, one tumor per animal was selected. In the case of being observed more than one malignant neoplasm per animal, the tumor with the most aggressive clinical and histopathological features (larger size, infiltrative growth, higher grade (Queiroga et al. 2010) was selected. According to the literature (Queiroga et al. 2010; 2014), the clinical stage of the animals was categorized into local (without lymph node involvement) and regional (metastasis at regional lymph nodes). For this classification, the TNM system (Owen and VPH/CMO/80.20 1980) was used where *T* describes the size of the primary tumor (higher diameter), *N* the presence (*N*+) or absence (*N*0) of lymph node metastasis and *M* the presence (*M*+) or absence (*M*0) of metastasis at distant organs. Of note that, tumor size (*T*1 < 3 cm; *T*2 ≥ 3 and <5 cm; *T*3 ≥ 5 cm) and skin ulceration were also two analyzed parameters. For the clinical follow-up, a physical examination, a radiological evaluation of the thorax and an abdominal ultrasound scan were performed in the animals 15 days after surgery and every 90 days thereafter for a minimum period of 730 days. The time of overall survival (OS) was calculated from the date of surgery to the date of animal death/euthanasia (due to advanced stages of the disease within 730 days) or to the date of the last clinical examination (dogs that survived more than 730 days). For the preparation of this manuscript, no procedure was carried out that was not strictly necessary for the treatment of each animal attended at AniCura CHV Porto Hospital Veterinário and Onevet Hospital Veterinário Porto, both located at Porto, Portugal and under clinical supervision of two clinicians (HG and LL). Only data collection was performed, without interfering with the clinical decisions taken in each case. Informed consent on the collection of samples and the clinical follow-up was obtained from each patient owner. This study was approved by the Scientific Council of the School of Agrarian and Veterinary Sciences, University of Trás-os-Montes and Alto Douro in 2011, as complying with Portuguese legislation for the protection of animals (Law No. 92/1995).

### 2.2. Histopathological examination

Collected samples were fixed in 10% buffered formalin and paraffin-embedded. Tissue sections with 4 μm thickness were stained with hematoxylin and eosin (HE) following routine methods. For diagnosis, each slide was evaluated according to the classification published by Davis–Thompson DVM Foundation (Zappulli et al. 2019). Furthermore, by using the method proposed by Peña and collaborators (Peña et al. 2013), the histological grade of malignancy (HGM) was evaluated for each sample. The presence of tumor necrosis, neoplastic intravascular emboli and regional lymph node involvement were also clinicopathological characteristics considered for the analysis. Tumor necrosis was evaluated as presence or absence, as previously described (Carvalho et al. 2016).

### 2.3. Antibodies

The following antibodies and conditions were used for immunohistochemistry assays: TGFβ [polyclonal antibody against TGFβ1 (sc-146), Santa Cruz Biotechnology, sc-146, Dallas, Texas, USA; 1:100], FoxP3 [(anti-mouse/human Foxp3 antibody, Clone eBio7979 (221D/D3), eBioscience, San Diego, USA; 1:100)], VEGF [(Clone JH121 (MA5-13182), Thermo Scientific, Waltham, MA USA; 1:100)], CD31 [(Clone JC70A Clone (IS610), Dako, Glostrup, Denmark; 1:20)].

### 2.4. Immunohistochemistry

FoxP3, TGFβ, VEGF and CD31 protein expression in tumors collected from the female dogs were evaluated by immunohistochemistry (IHC). IHC for FoxP3 was performed using a polymeric labeling methodology (Novolink Polymer Detection System; Novocastra, Newcastle, UK) whereas for TGFβ, VEGF and CD31 a streptavidin–biotin–peroxidase complex method with the Ultra Vision Detection System kit (Lab Vision Corporation, Fremont, CA, USA) was used, as previously described by us (Carvalho et al. 2016). Briefly, deparaffinized and rehydrated slides were submitted to microwave antigen retrieval for 3 cycles of 5 min at 750 W with 0.01 M citrate buffer (pH 6.0). Followed 20 min cooling at room temperature, sections were incubated overnight with the primary antibodies at 4 °C. The antibody reactions were visualized with the chromogen 3,3′-diaminobenzidine tetrachloride (DAB; Dako, Denmark). The slides were counterstained with Gill’s hematoxylin, dehydrated, cleared and mounted. For each immunoreaction, positive and negative controls were included. As negative control, the primary antibody was replaced with an irrelevant isotype-matched antibody. As positive control for TGFβ, intestine sections were used. In the case of FoxP3, sections of canine lymph nodes were used. Liver section and dog angiosarcoma were used for VEGF and CD31, respectively.

### 2.5. TGFβ, FoxP3, VEGF and CD31 staining evaluation

Intratumoral FoxP3, VEGF and CD31 (PECAM-1) used for determining microvascular density, were evaluated using a well-established method already applied in other studies by our group (Queiroga et al. 2011; Carvalho et al. 2013; Raposo et al. 2015; Carvalho et al. 2016).

TGFβ immunoreactivity was evaluated in the intratumoral area by two independent experts that ­analyzed the entire slides (×200 magnification) using an immunohistochemical semiquantitative method adapted from previous published study (Ding et al. 2016). The method final score was achieved by the product of the percentage of positive cells (immunolabelling extension) and staining intensity. The percentage of positive cells was scored as 0 (0% positive cells), 1 (<10% positive cells), 2 (10–50% positive cells), 3 (51–80% positive cells), or 4 (>80%) whereas the staining intensity was scored as 1 (weakly stained), 2 (moderately stained), and 3 (strongly stained). Low TGFβ class was considered if the product of multiplication between staining intensity and the percentage of positive cells was ≤ 6. A final immunohistochemical score > 6 indicates a high TGFβ class.

### 2.6. Statistical analysis

Statistical analysis was performed using SPSS software version 27.0 (Statistical Package for the Social Sciences, Chicago, IL, USA). Categorical variables were analyzed using the Chi-square test, while continuous variables were assessed through Analysis of Variance (ANOVA) with Tukey’s multiple means comparison. Correlations were evaluated using Pearson’s correlation test for parametric variables and Spearman’s correlation test for nonparametric variables. Survival curves were constructed using the Kaplan–Meier method with mean values as the cutoff, and differences in survival were analyzed using the log-rank test. Multivariate survival analysis was conducted using Cox regression analysis, including all variables simultaneously *via* the enter method. All tests were assessed at a 95% confidence level (*p* < 0.05).

## 3. Results

### 3.1. Clinicopathological data

Most of the tumors included in this study were histologically classified as tubulopapillary carcinomas (*n* = 31). Others include 8 solid carcinomas, 12 complex carcinomas, 3 anaplastic carcinomas and 13 carcinosarcomas. Twenty-eight tumors had lymph node metastasis. Twenty-one tumors presented intravascular neoplastic emboli (31.3%). The HGM was classified as I (*n* = 17, 25.4%), II (*n* = 18, 26.8%), or III (*n* = 32, 47.8%).

### 3.2. Expression of TGFβ, FoxP3, VEGF and CD31 in malignant CMT

**Figure 1.**
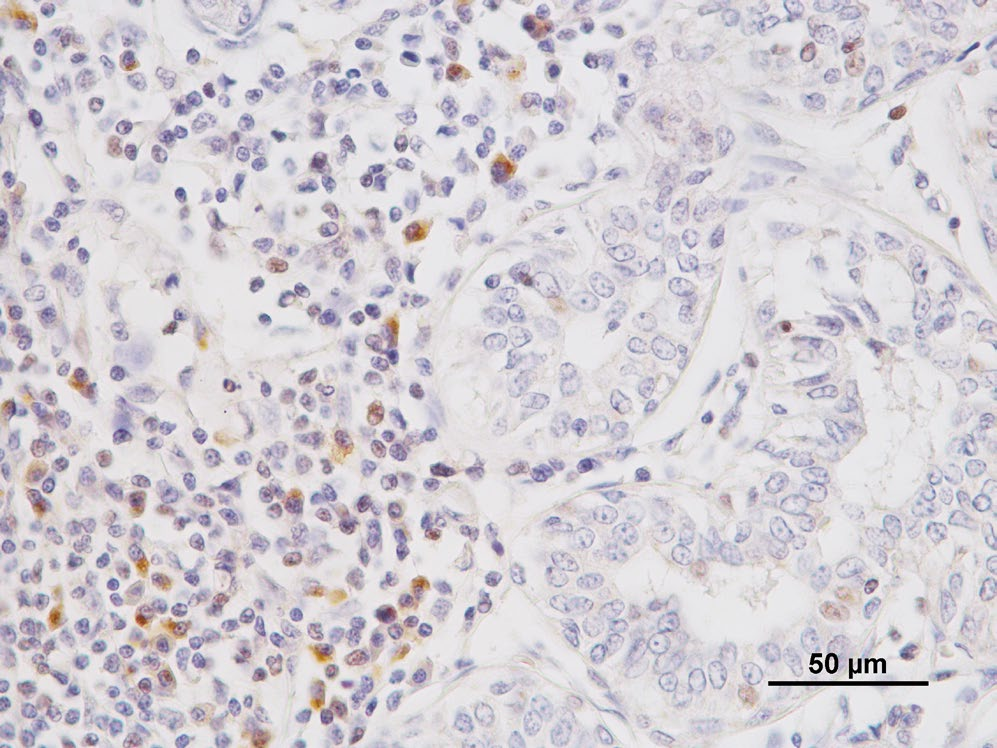
Foxp3^+^ expression in the nuclei of lymphoid cells infiltrating a tubulopapillary carcinoma, Scale bar = 50 µm.

Part of FoxP3, VEGF and CD31 cases included in this work were already used in a study published by our team where the staining patterns observed in the samples were already described (Carvalho et al. 2016). The mean number (±SE) of intratumoral FoxP3^+^ regulatory T cells was 73.88 ± 6.585 (range 19–267; Figure 1). The mean number (±SE) of total neovessels was 39.01 ± 2.562 (range 6–106).

**Figure 2.**
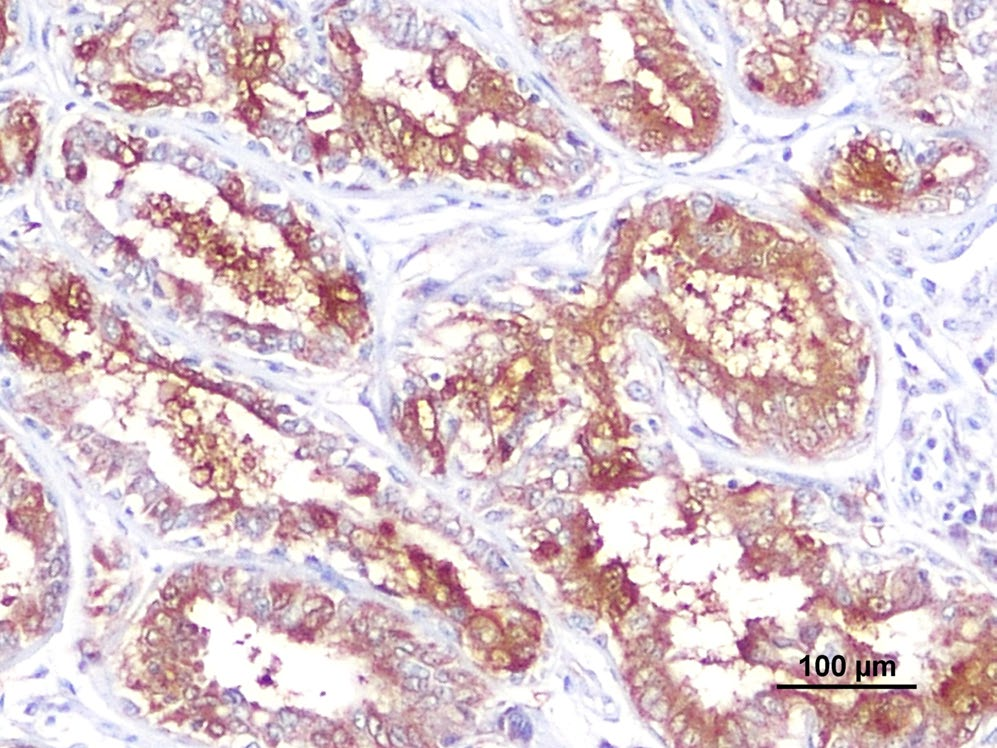
Tubulopapillary carcinoma with high expression of TGFβ, note the most evident diffuse cytoplasmic staining, with prominence of the cytoplasmic membrane, Scale bar = 100 µm.

The anti-TGFβ antibody had high affinity for tumor epithelial cells. TGFβ immunoexpression was predominantly a diffuse or granular cytoplasmic staining, most evident in the cytoplasm of the ductal epithelium, with prominence of the cytoplasmic membrane (Figure 2). Regarding TGFβ percentage of immunolabelled cells, 10 cases showed extension 1 (<10% positive cells), 18 cases showed extension 2 (10–50% positive cells), 23 cases and 16 cases demonstrated extension 3 (51–80% positive cells) and 4 (>80%) respectively. For TGFβ labelling intensity, there was also a relatively homogeneous distribution between moderate (40.3%, *n* = 27) and strong labelling (35.8%, *n* = 24), whereas tumors with weak intensity (23.9%, *n* = 16) were less frequent.

### 3.3. Associations of TGFβ immunostaining with clinicopathological features

Our analysis has identified a striking association between the presence of aggressive disease and high expression of TGFβ. Tumors with higher levels of TGFβ were associated with skin ulceration (*p* = 0.018), tumor necrosis (*p* = 0.024), high HGM (*p* < 0.001), presence of neoplastic intravascular emboli (*p* < 0.001) and presence of lymph node metastasis (*p* < 0.001). Table 1 highlights all the results described above.

**Table 1.** Relationship between TGFβ class and clinicopathological parameters in malignant canine mammary tumors.

Clinicopathological parameters	Low TGFβ	High TGFβ	Adjusted OR (95% CI)*	*p*
	*N*	*n*		
Tumor size				
T1 < 3cm	19	6		
T2 ≥ 3 cm and <5cm	12	7		NS
T3 ≥ 5 cm	11	10		
Skin ulceration				
Absent	35	13	Reference^a^	**0.018**
Present	8	11	3.74 (1.21–11.24)	
Histological type				
Tubulopapillary C.	22	9		
Solid C.	4	4		
Complex C.	9	3		NS
Anaplastic C.	0	3		
Carcinosarcoma	5	8		
Tumor necrosis				
Absent	23	6	Reference^a^	**0.024**
Present	20	18	3.45 (1.14–10.37)	
Histological grade of malignancy				
I	16	1	Reference^a^	**<0.001**
II	17	1	0.94 (0.05 a 16.34)	
III	10	22	35.2 (4.08 a 303.44)	
Neoplastic intravascular emboli				
Absent	36	10	Reference^a^	**<0.001**
Present	7	14	7.20 (2.28–22.65)	
Lymph node metastasis				
Absent	34	5	Reference^a^	**<0.001**
Present	9	19	14.35 (4.2–49.06)	

*n* number of samples; *p* statistical significance; *C* carcinoma; NS not significant, OD odds ratio; CI confidence interval.

^a^ Reference category.

### 3.4. Correlation between TGFβ, FoxP3, VEGF and CD31 immunoexpression

The levels of TGFβ were positively correlated with intratumoral FoxP3 (*r* = 0.719; *p* < 0.001), VEGF (*r* = 0.378; *p* = 0.002) and CD31 (*r* = 0.511; *p* < 0.001). In this study three classes were considered: TGFβ/FoxP3, TGFβ/VEGF and TGFβ/CD31. Each class was divided in three categories: 1) low immunoreactivity for both markers; 2) low immunoreactivity for one marker and high for other and 3) high immunoreactivity for both markers.

### 3.5. Association of TGFβ/VEGF class with intratumoral FoxP3 and MVD in malignant CMT

**Figure 3.**
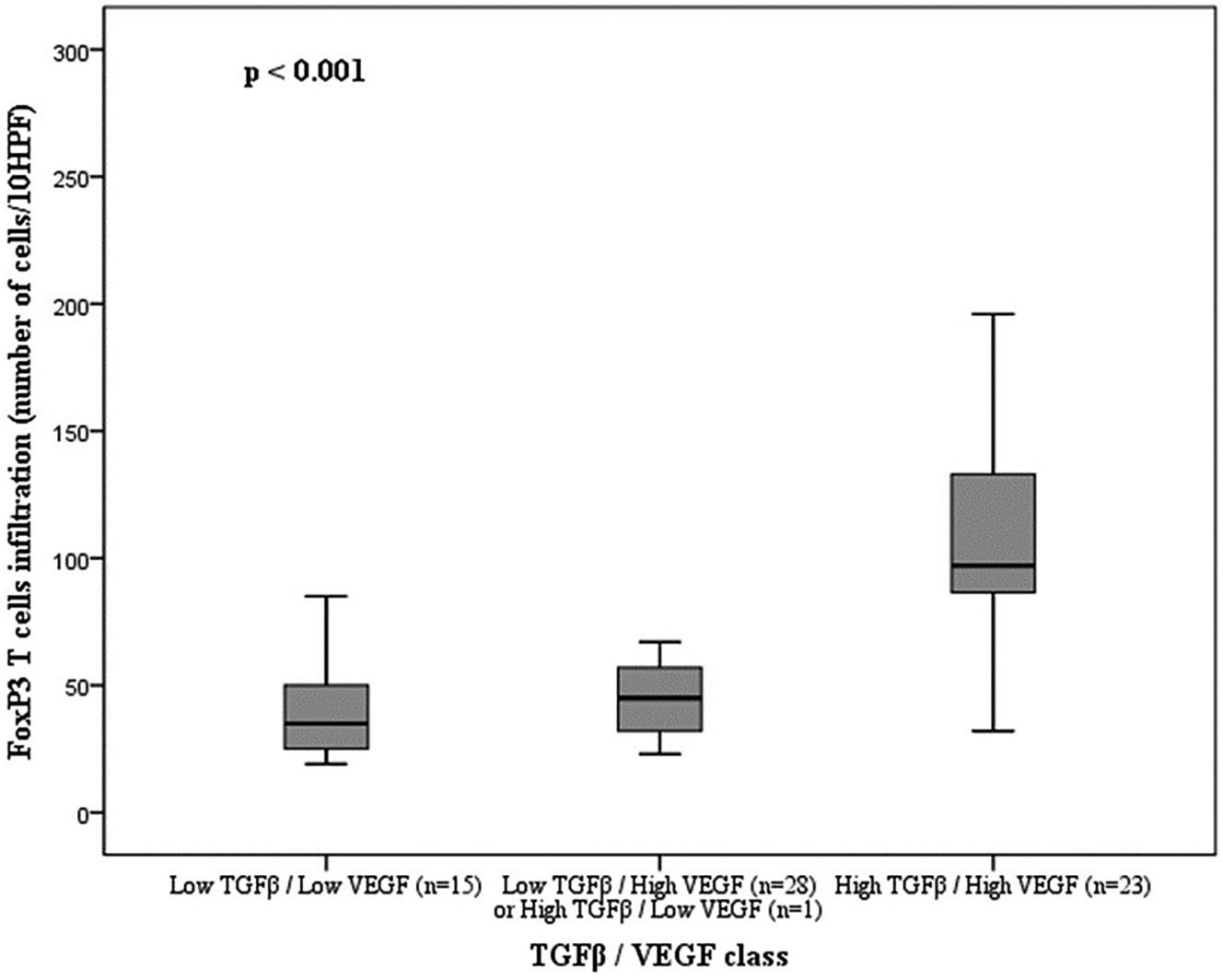
FoxP3 T cells distributed according to the TGFβ/VEGF class and respective value of statistical significance for the ANOVA test.

The FoxP3-positive T cells in tumors with concurrent high TGFβ/VEGF immunoexpression (*n* = 23; mean 118.26 ± 12.535; range: 32–267) were higher than FoxP3-positive T cells in tumors with low immunoexpression for both markers (*n* = 15; mean 41.80 ± 5.446; range: 19–85). The FoxP3 expression was also higher in tumors with high immunoexpression of only one of the markers [tumors TGFβ low/VEGF high (*n* = 28) or tumors TGFβ high/VEGF low (*n* = 1)] (*n* = 29; mean 55.28 ± 6.586; range: 23–186), compared to tumors with low expression for both markers (*p* < 0.001; Figure 3).

**Figure 4.**
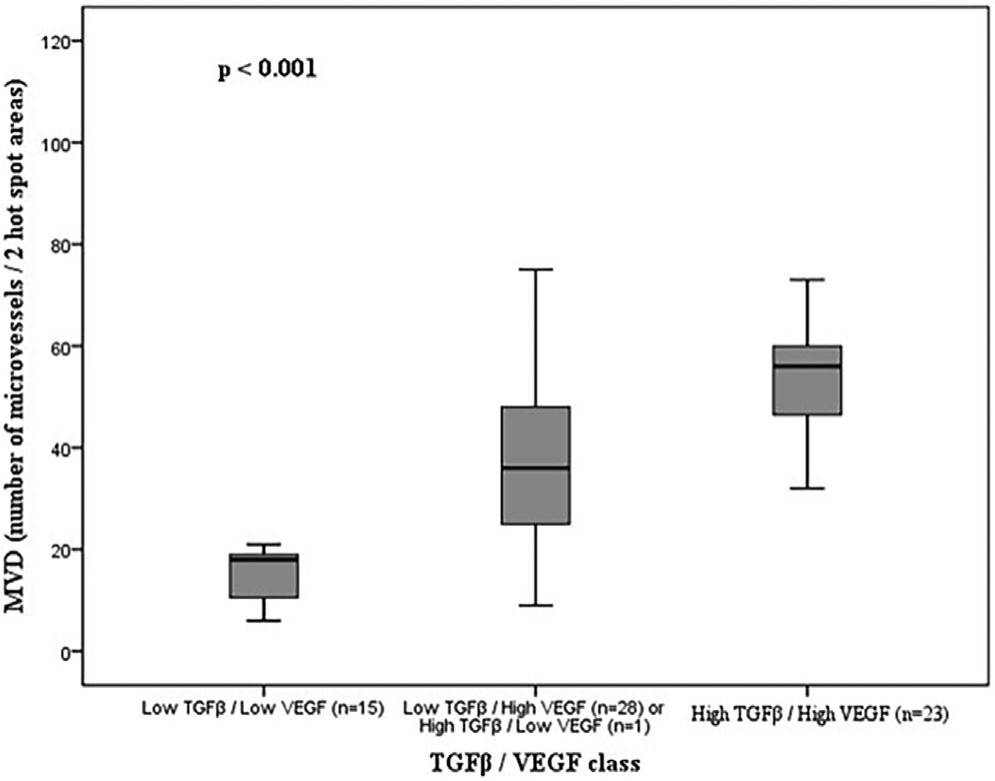
Association of MVD (number of microvessels) distributed according to the TGFβ/VEGF class and respective value of statistical significance for the ANOVA test.

Similar results were observed for MVD. Tumors with high TGFβ/VEGF immunoexpression (*n* = 23; mean 53.70 ± 2.872; range: 22–89) showed higher values of microvessels compared with tumors with low immunoexpression for both markers (*n* = 15; mean 15.40 ± 1.337; range: 6–21). The mean MVD was also higher in tumors with high immunoexpression of only one of the markers [tumors TGFβ low/VEGF high (*n* = 28) or tumors TGFβ high/VEGF low (*n* = 1)] (*n* = 29; mean 39.59 ± 3.705; range: 9–106), compared to tumors with low expression for both markers (*p* < 0.001; Figure 4).

### 3.6. Relationship of TGFβ/FoxP3, TGFβ/VEGF and TGFβ/CD31 classes with clinicopathological variables of tumor aggressiveness

Tumors with concurrent high expression of TGFβ/FoxP3, TGFβ/VEGF and TGFβ/CD31 markers were associated with parameters of tumor malignancy: high HGM (*p* < 0.001 for TGFβ/FoxP3, TGFβ/VEGF and TGFβ/CD31), presence of neoplastic intravascular emboli (*p* < 0.001 for TGFβ/FoxP3 and TGFβ/CD31; *p* = 0.001 for TGFβ/VEGF) and presence of lymph node metastasis (*p* < 0.001 for TGFβ/FoxP3, TGFβ/VEGF and TGFβ/CD31). More information is provided in Table 2.

**Table 2.** Relationship of TGFβ/FoxP3, TGFβ/VEGF and TGFβ/CD31groups with clinicopathological variables of tumor aggressiveness.

	Variables of tumor aggressiveness									
	HGM				Neoplastic intravascular emboli			Lymph node metastasis		
	I	II	III	*p*	Absent	Present	*p*	Absent	Present	*p*
Molecular markers										
TGFβ/FoxP3										
Low TGFβ/low FoxP3	15	16	6	**<0.001**	35	2	**<0.001**	32	1	**<0.001**
Low TGFβ/high FoxP3 or high TGFβ/low FoxP3	2	2	5		3	6		4	5	
High TGFβ/high FoxP3	0	0	21		8	13		3	18	
TGFβ/VEGF										
Low TGFβ/low VEGF	6	8	1	**<0.001**	14	1	**0.001**	13	2	**<0.001**
Low TGFβ/high VEGF or high TGFβ/low VEGF	11	9	9		23	6		22	7	
High TGFβ/high VEGF	0	1	22		9	14		4	19	
TGFβ/CD31										
Low TGFβ/low CD31	12	15	3	**<0.001**	28	2	**<0.001**	27	3	**<0.001**
Low TGFβ/high CD31 or high TGFβ/low CD31	5	3	9		11	6		9	8	
High TGFβ/high CD31	0	0	20		7	13		3	17	

*n*, number of samples; *p*, statistical significance; NS, not significant.

### 3.7. Follow-up study

In this study, tumors of histological types carcinosarcoma, anaplastic carcinoma and solid carcinoma (*p* = 0.002), larger size (*p* = 0.011), presence of tumor necrosis (*p* = 0.002), neoplastic intravascular emboli (*p* < 0.001), lymph node metastasis (*p* < 0.001), high HGM (*p* < 0.001) and higher levels of CD31 (*p* = 0.001), VEGF (*p* = 0.02) and FoxP3 (*p* < 0.001), were associated with lower OS time. All these findings are summarized in Table 3.

**Table 3.** Univariate overall survival analysis for the clinicopathological variables included in the study.

	Overall survival time (days)					
	*N*	Mean (d)	Confidence interval (95%)	Median (d)	Confidence interval (95%)	*p*
Variables						
Tumor size						
T1 < 3cm	25	634	557.338–711.862	a*	–	**0.011**
T2 ≥ 3cm and < 5 cm	19	447	338.076–557.608	350	321.561–378.439	
T3 ≥ 5 cm	21	444	331.459–557.494	407	182.709–631.291	
Skin ulceration						
Absent	48	547	478.953–615.281	a*	–	0.188
Present	19	452	336.640–567.886	420	317.621–522.379	
Histological type						
Tubulopapillary C.	31	580	499.360–661.400	a*	–	**0.002**
Solid C.	9	371	223.659–518.785	356	338.469–373.531	
Complex C.	12	664	580.695–748.805	a*	–	
Anaplastic C.	2	319	12.710–625.290	98	–	
Carcinosarcoma	13	362	237.172–487.443	329	179.861–478.139	
Tumor necrosis						
Absent	29	630	563.644–697.253	a*	–	**0.002**
Present	38	435	352.831–579.868	356	254.627–457.189	
Histological grade of malignancy						
1	17		b*		b*	**<0.001**
2	18					
3	32					
Neoplastic intravascular emboli						
Absent	46	622	563.243–682.540	a*	–	**<0.001**
Present	21	295	219.529–579.868	240	186.170–293.830	
Lymph node involvement						
Absent	39	666	615.148–717.762	a*	–	**<0.001**
Present	28	316	242.176–579.868	240	102.580–377.420	
CD31						
Low	34	618	544.853–691.536	a*	–	**0.001**
High	33	419	337.704–500.720	356	299.729–412.271	
VEGF						
Low	16	666	592.945–739.847	*	–	**0.02**
High	51	474	403.927–544.740	420	–	
FOXP3						
Low	40	647	591.077–702.930	a*	–	**<0.001**
High	27	332	250.627–413.965	270	91.894–448.106	
TGFβ extension						
1	10	668	553.002–783.198	a*	–	**<0.001**
2	18	588	486.596–690.367	a*	–	
3	23	594	515.347–673.696	a*	–	
4	16	244	163.517–325.733	201	155.920–246.080	
TGFβ intensity						
1	16	691	617.893–764.732	a*	–	**0.004**
2	27	493	397.221–588.802	630	–	
3	24	437	340.034–534.132	360	255.578–464.422	
TGFβ score						
Low	43	627	567.129–687.372	a*	–	**<0.001**
High	24	328	244.716–412.034	270	159.557–380.423	
TGFβ score/FOXP3						
Low TGFβ/low FoxP3	37	654	599.408–709.900	a*	–	**<0.001**
Low TGFβ/high FoxP3 or high TGFβ/low FoxP3	9	490	313.635–667.032	a*	–	
High TGFβ/high FoxP3	21	296	218.730–373.651	270	112.996–427.004	
TGFβ score/VEGF						
Low TGFβ/low VEGF	15	662	584.907–740.515	a*	–	**<0.001**
Low TGFβ/high VEGF or high TGFβ/low VEGF	29	612	533.633–691.884	a*	–	
High TGFβ/high FoxP3	23	310	230.924–390.902	270	105.659–434.341	
TGFβ score/CD31						
Low TGFβ/low CD31	30	644	577.065–711.826	a*	–	**<0.001**
Low TGFβ/high CD31 or high TGFβ/low CD31	17	548	427.329–670.435	a*	–	
High TGFβ/high CD31	20	309	232.039–387.161	270	50.865–489.135	

a* Not reached; b* non-computable as all the cases are censored in category 1; *n* number of animals; d days.

**Figure 5.**
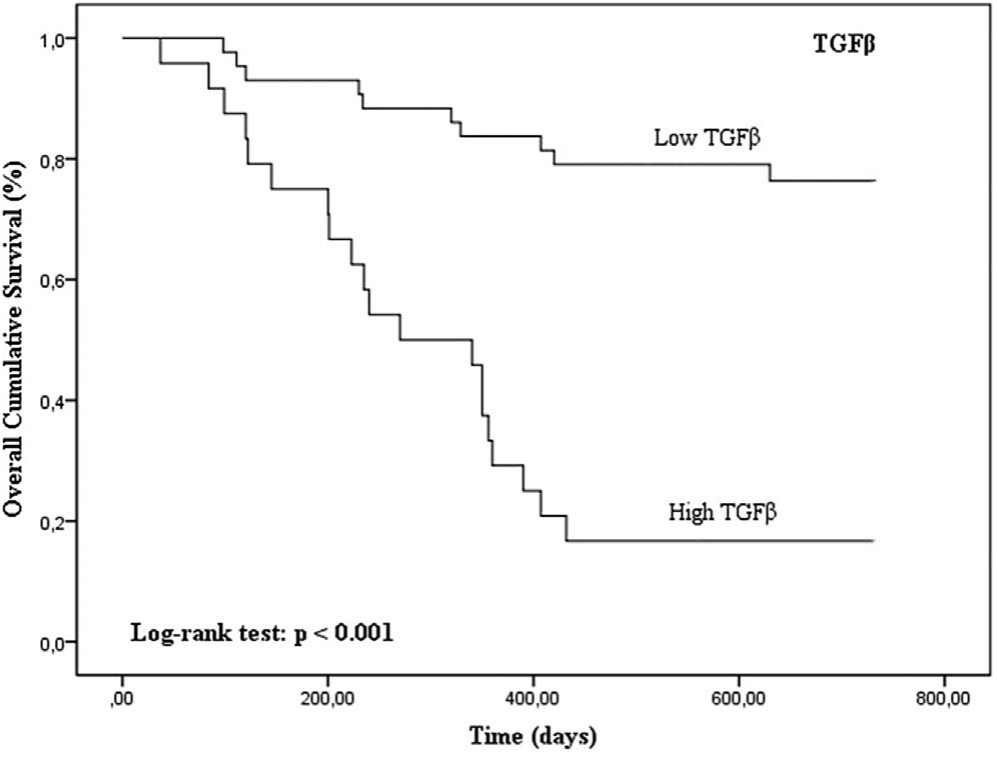
Kaplan–Meier OS curves comparing TGFβ categories in 67 dogs with malignant mammary tumors.

**Figure 6.**
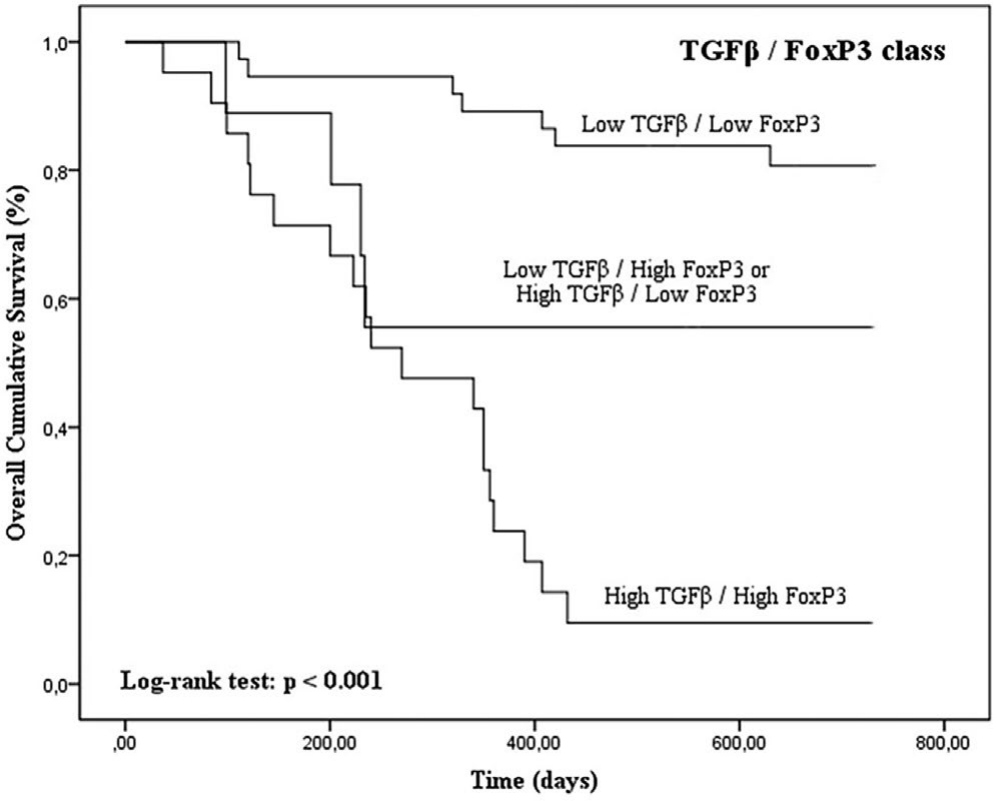
Kaplan–Meier OS curves comparing TGFβ/FoxP3 categories in 67 dogs with malignant mammary tumors.

**Figure 7.**
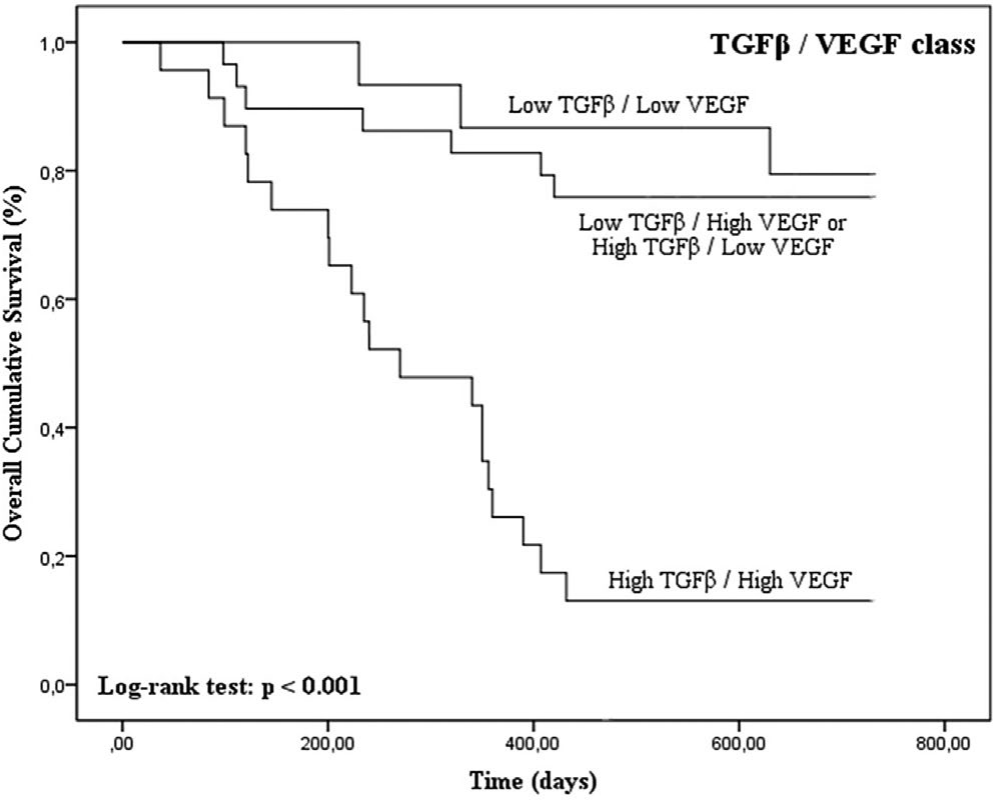
Kaplan–Meier OS curves comparing TGFβ/VEGF categories in 67 dogs with malignant mammary tumors.

**Figure 8.**
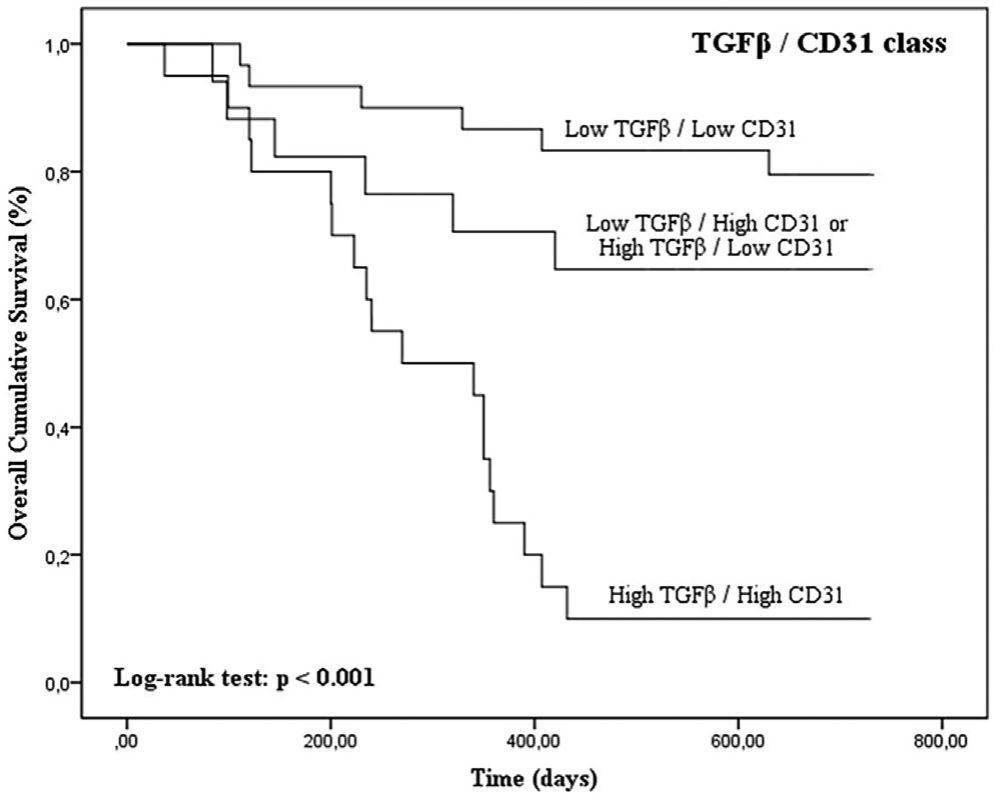
Kaplan–Meier OS curves comparing TGFβ/CD31 categories in 67 dogs with malignant mammary tumors.

Tumors with high TGFβ levels and with concurrent high expression of TGFβ/FoxP3, TGFβ/VEGF and TGFβ/CD31 were associated with shorter OS time (*p* < 0.001 for TGFβ, TGFβ/FoxP3, TGFβ/VEGF and TGFβ/CD31 in Kaplan–Meier curves; Figures 5–8; Table 3).

The presence of lymph node metastasis retained the association with shorter OS in multivariate Cox regression analysis, arising as an independent predictor of poor prognosis [Hazard ratio (95% CI): 11.033 (1.358–89.653); *p* = 0.025].

## 4. Discussion

This study primarily explored the immunoexpression of TGFβ, FoxP3, VEGF, and CD31 in malignant CMT and their associations with tumor clinicopathological features. We found that TGFβ immunoexpression was associated with aggressive tumor characteristics such as skin ulceration, tumor necrosis, higher HGM, neoplastic intravascular emboli, and lymph node metastasis. Additionally, a positive correlation was observed between TGFβ, FoxP3, VEGF, and CD31.

TGFβ demonstrates a dual role in malignant tumor development process. During the early stages of carcinogenesis, TGFβ acts as a tumor suppressor, regulating negatively cellular proliferation. However, with the development of malignant tumor, the TGFβ role changes toward a tumor promoter, mediating tumor cells proliferation, migration and invasion (Dumont and Arteaga 2000; Moses and Barcellos-Hoff 2011; Ding et al. 2016; Colak and Ten Dijke 2017).

Findings suggest that the dysregulation of TGFβ pathways in tumors induce signal reprogramming, allowing cancer cells to mimic normal functions to guarantee their subsistence. In fact, recent studies have demonstrated that high levels of TGFβ expression have a close association with several human malignancies (Coban et al. 2007; Minamiya et al. 2010; Stojnev et al. 2019; Perez et al. 2020; Torrealba et al. 2020), including breast cancer (Bao et al. 2009; Lang et al. 2014; Juang et al. 2016; Huang et al. 2021; Niu et al. 2021).

In human breast cancer high levels of TGFβ are observed in advanced carcinomas, and have been correlated with disease progression and worse clinical outcomes (Gorsch et al. 1992; Buck et al. 2004; Bao et al. 2009; Juang et al. 2016; Huang et al. 2021). TGFβ produced by tumor cells may act in a paracrine mode on tumor stromal cells, tumor neovessels and immune cells, contributing to tumor immunosuppression, angiogenesis and progression (Dumont and Arteaga 2000; Lang et al. 2014; Niu et al. 2021).

In veterinary literature, to the best of our knowledge, the prognostic value and the role that TGFβ may have on CMT immunosuppression and angiogenesis were not investigated yet. The findings of our work are in accordance with recent literature in human breast cancer (Gorsch et al. 1992; Bao et al. 2009; Lang et al. 2014; Ding et al. 2016; Juang et al. 2016) and suggests a link between TGFβ and more aggressive tumor phenotypes, reflecting its involvement in CMT malignant transformation. In veterinary field, one study demonstrated using a CMT cell line that TGFβ prompt an induction of the mesenchymal marker vimentin, increasing the invasiveness capacity of tumor cells, a crucial step in metastasis formation. Interestingly, this induction is reversed in a phenomenon similar to the mesenchymal–epithelial ­transition (the reverse phenomenon of epithelial–mesenchymal transition) after prolonged stimulation with TGFβ. This is a beneficial effect for the formation of new tumor masses at the side of metastatic lesions (Yoshida et al. 2013). Another study also showed a higher immunohistochemical expression of matrix metalloproteinase‐9 (MMP-9) and TGFβ in malignant CMT in comparison with benign ones. Additionally, *in vitro* activation of TGFβ/SMAD pathways induced an overexpression of MMP‐9 in the breast cancer cell lines and an increase in breast cells malignancy (Dong et al. 2019). These results corroborate our work and the lack of additional studies in CMT hampers more concise comparisons. Our data demonstrated also that TGFβ levels showed a strong positive correlation with intratumoral FoxP3, VEGF, and CD31 levels.

Concordantly with our findings, in human breast cancer TGFβ has an important role on tumor microenvironment switch, promoting increased angiogenic activity and suppressed immune surveillance, contributing to tumor development, progression and poor clinical outcome (Donovan et al. 1997; Gupta et al. 2007; Petersen et al. 2010; Ding et al. 2016; Juang et al. 2016). The TGFβ in breast tumor sites acts as an important immunosuppressant repressing effector T cells anti-tumor activity (Padua and Massagué 2009; Stüber et al. 2020; Lainé et al. 2021). Additionally, TGFβ signaling in T cells participates in the expression and the stabilization of transcription factor FoxP3. The increasingly high concentrations of TGFβ secreted by tumor cells induce FoxP3 expression in peripheral CD4^+^CD25^–^T cells and their precursors, rendering them inactive (Chen and Konkel 2010; Principe et al. 2014). This occurrence is clinically relevant since the enrichment of CD4^+^CD25^+^FoxP3^+^Treg cells in human mammary tumors is associated with poor prognosis (Gupta et al. 2007; Kajal et al. 2021; Lainé et al. 2021). Moreover, Treg cells increase the TGFβ effects creating a positive auto-regulatory loop of TGFβ signaling in CD4^+^CD25^–^T cells that possibly stabilizes their regulatory phenotype (Fantini et al. 2004). FoxP3 Treg cells, in this process, needs greater attention, not only for being an important source of TGFβ but also for directly instructing cancer cells by secreting TGFβ (Jensen-Jarolim et al. 2015). In humans the TGFβ and FoxP3 common signaling pathways have a crucial impact in several phases of mammary carcinogenesis, including tumor angiogenic switch (Gupta et al. 2007; Padua and Massagué 2009; Chen and Konkel 2010; Kajal et al. 2021; Lainé et al. 2021). Equally to our results, data in human breast cancer demonstrated that intratumoral FoxP3 was correlated with levels of TGFβ, VEGF and tumor microvessel density (Gupta et al. 2007; Lainé et al. 2021). TGFβ and FoxP3 are reported to regulate tumor new blood vessels formation by a combination of responses that increase the production of VEGF (Donovan et al. 1997; Gupta et al. 2007; Petersen et al. 2010; Kajal et al. 2021).

In dog mammary tumors it was demonstrated that Treg cells may contribute to increased angiogenesis (Carvalho et al. 2016). Another study showed that FoxP3^+^CD4^+^T cells in dogs could be expanded *in vitro* after the addition of TGFβ and IL-2 and by tumor cell receptor activation (Biller et al. 2007). However, to the best of our knowledge, this is the first study that demonstrate the prognostic value of TGFβ. Interestingly our results suggest that in CMT may exist an autocrine/paracrine TGFβ/FoxP3 signaling loop. TGFβ and Treg cells common pathways provides the tumor with a mechanism that facilitate evasion of immune surveillance and prompt the VEGF-dependent angiogenesis, contributing to CMT progression and aggression.

## 5. Conclusion

In our study, tumors with concurrent high expression of TGFβ with FoxP3, VEGF, or CD31 were significantly associated with clinicopathologic factors typically related to clinical aggressiveness (high HGM, presence of vascular emboli and nodal metastasis), and linked to shorter OS times. Despite these relevant associations between the prognosis and immunologic and angiogenic markers, the lymph node metastasis was the single independent predictor of poor prognosis in this case series of CMTs.
